# Successful Outcome of Stage IB3 Cervical Cancer Treated With Neoadjuvant Chemotherapy Followed by Vaginal Radical Trachelectomy: A Case Report

**DOI:** 10.7759/cureus.71626

**Published:** 2024-10-16

**Authors:** Masato Tamate, Motoki Matsuura, Masayuki Someya, Tasuku Mariya, Shinichi Ishioka, Tsuyoshi Saito

**Affiliations:** 1 Obstetrics and Gynecology, Sapporo Medical University, Sapporo, JPN

**Keywords:** childbearing woman, fertility-sparing surgery, neoadjuvant chemoradiotherapy, radical trachelectomy, uterine cervical cancer

## Abstract

Vaginal trachelectomy, which involves resecting the cervix and its parametrium, is a fertility-sparing option for the treatment of early-stage cervical cancer. Although no consensus has been reached on whether simple or radical trachelectomy is preferable, the vaginal approach is typically avoided for tumors larger than 2 cm due to concerns about recurrence. However, some evidence suggests that fertility preservation may still be viable for select patients with larger tumors. This case report describes a woman with bulky cervical cancer who wished to preserve her fertility. After undergoing neoadjuvant chemotherapy (NAC) and vaginal radical trachelectomy (VRT), she achieved a favorable oncological and perinatal outcome, successfully giving birth to a near-full-term baby. The report outlines the patient's management before, during, and after the procedure, including perinatal care. While careful selection of candidates is crucial, accumulating case reports and future trials are expected to shed more light on this treatment approach.

## Introduction

In some countries, the incidence of cervical cancer is declining due to the introduction of vaccines [1]. However, cervical cancer remains the fourth most common oncological disease among women worldwide. Nearly 40% of women diagnosed with cervical cancer are between the ages of 20 and 44, particularly in Japan, where the vaccine introduction was delayed [2]. The standard treatment for International Federation of Gynecology and Obstetrics (FIGO) 2018 stage 1B2-1B3 cervical cancer involves radical hysterectomy and pelvic lymph node dissection. For women wishing to preserve fertility, trachelectomy is an option according to the National Comprehensive Cancer Network (NCCN) guidelines [3]. However, conservative management is rarely offered for tumors >2 cm due to the higher risk of recurrence, particularly after primary radical trachelectomy via the vaginal approach. Abdominal radical trachelectomy (ART) is associated with a lower recurrence rate than vaginal radical trachelectomy (VRT), although it significantly lowers pregnancy rates [4]. Another option is NAC followed by trachelectomy and pelvic lymph node dissection (PLND). With various surgical approaches, including abdominal, laparoscopic, robotic, and vaginal, as well as simple or radical procedures, the optimal management of fertility-preserving surgery for tumors >2 cm remains controversial.

This case report discusses the successful outcome of a woman with a strong desire to preserve fertility, who was treated with NAC followed by VRT. She conceived naturally six months post-surgery and delivered via cesarean section.

## Case presentation

A 27-year-old woman, nulligravida with a body mass index of 19 kg/m², presented with abnormal vaginal bleeding that lasted about a month but no other symptoms. Her menstrual cycle was normal, and she had no gynecologic history. She had never been screened for cervical cancer, and both she and her husband wanted to have children. A biopsy of the cervix confirmed squamous cell carcinoma. Colposcopy, pelvic MRI, and CT revealed a 40 × 35 × 25 mm cervical tumor without parametrial invasion, lymph node involvement, or distant metastasis. Serum SCC was elevated at 7.1. She was diagnosed with cervical cancer cT1b3N0M0, FIGO stage IB3.

**Figure 1:**
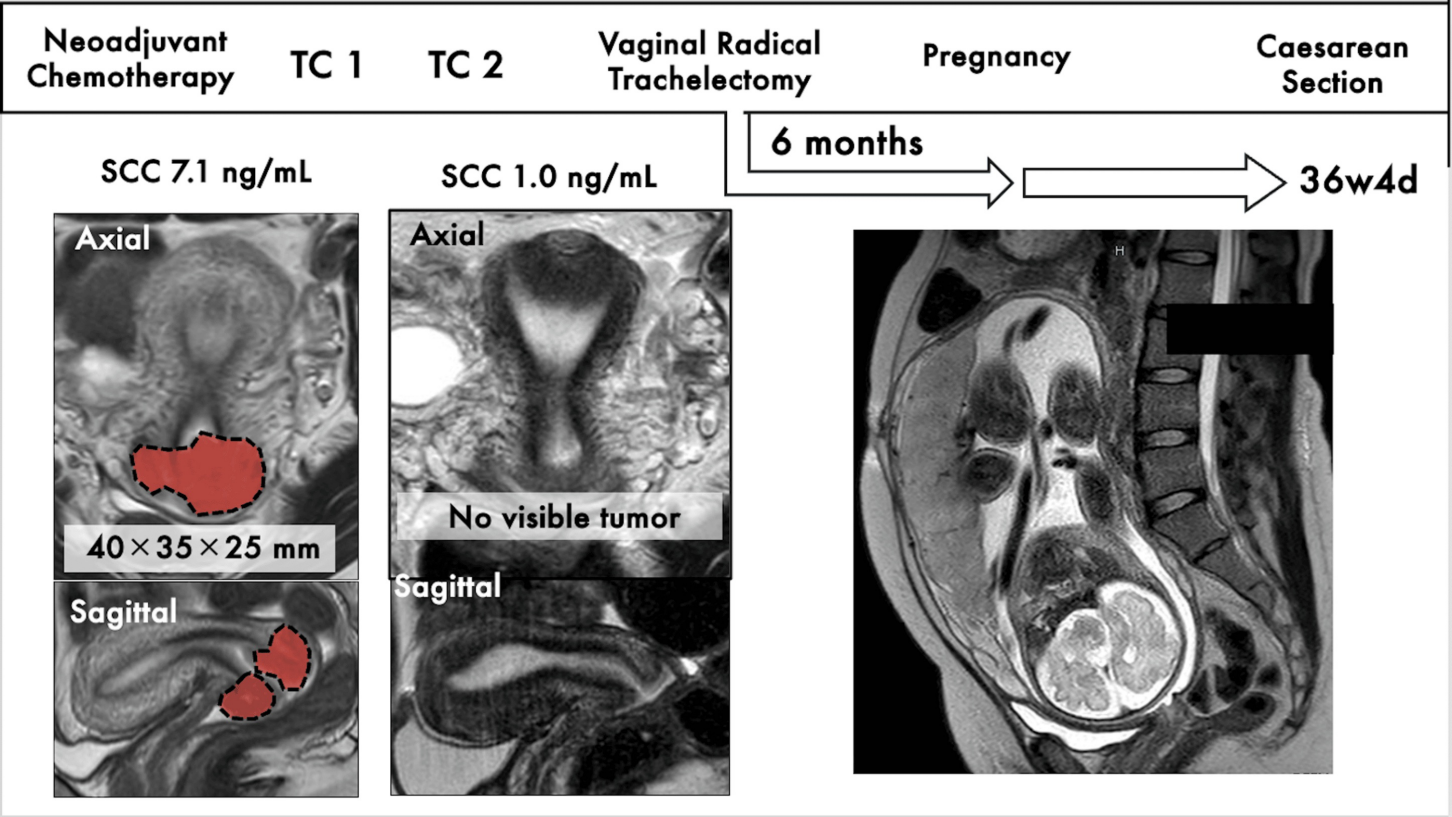
Timeline from initial treatment to delivery Initial examination revealed a 4 cm tumor with elevated tumor markers. However, preoperative chemotherapy resulted in a complete response, followed by vaginal radical trachelectomy (VRT) and laparoscopic lymph node dissection. Six months after surgery, the patient conceived and gave birth naturally.

Radical hysterectomy was recommended as the standard treatment, but the patient and her family opted for fertility preservation. After discussion, NAC with paclitaxel 175 mg/m^2^ and carboplatin AUC6 (TC) was initiated. After two cycles, the tumor size decreased to 20 × 15 × 10 mm. Figure 1 shows a timeline of her treatment including the pre- and post-chemotherapy images. Despite the risks, the patient chose to proceed with VRT and PLND. One month after the second chemotherapy cycle, the SCC level became negative, and the tumor was undetectable. Surgery proceeded as follows: laparoscopic lymph node dissection was performed, and then VRT was conducted using techniques described in previous studies [5]. The operation lasted four hours and 48 minutes with 80 g of blood loss. Histopathology revealed a non-keratinized squamous cell carcinoma (ypT1a1), with 2.0 mm stromal invasion and negative margins.

**Figure 2:**
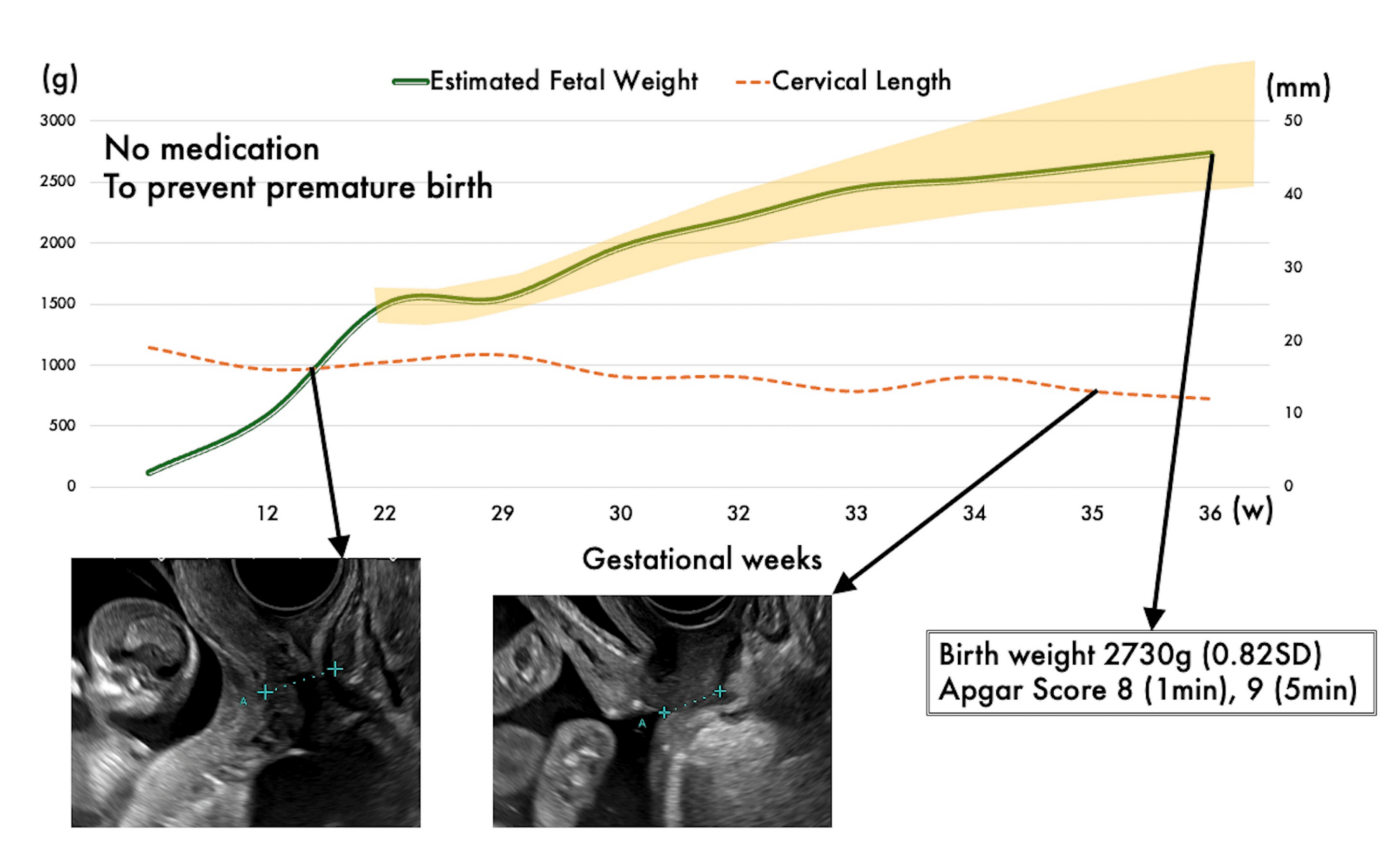
Fetal growth and sonography of cervical length Fetal growth was steady, remaining around 0 SD on the standard Japanese fetal growth curve. In addirion, although the cervix was shortened, there was no evidence of uterine contractions or cervical dilation that required medication.

Pap smears and SCC tests were conducted monthly post-surgery, and there was no recurrence at six months. The patient conceived naturally after eight months. Figure 2 shows the pregnant term. Despite a shortened cervix (15-19 mm), there were no signs of preterm labor. She had a smooth pregnancy and did not require any medication to prevent preterm labor. MRI scans showed no evidence of recurrent disease. Measurements and Pap smears were conducted more frequently than usual. She delivered a healthy baby at 36 weeks and four days via cesarean section. There were no signs of malignancy or adhesions. The patient is currently free of disease and is considering a second pregnancy. 

## Discussion

Many studies have demonstrated the safety and efficacy of trachelectomy in early-stage cervical cancer. The NCCN guidelines do not strictly exclude patients with tumors >2 cm, although European guidelines regard fertility-sparing surgery for such cases as experimental [3,6]. This case highlights the effectiveness of NAC-TC, followed by VRT, in achieving both oncological control and successful pregnancy. However, if the surgical margins had been positive or if lymph node metastasis had been detected, standard treatment would have been necessary, as discussed with the patient.

Recent trials have sparked debate over the optimal surgical approach. Compared to ART (including laparoscopic and robotic systems), VRT offers easier resectable margins but involves a shorter extent of parametrium resection. This may result in outcomes similar to those of modified radical hysterectomy. Consequently, VRT has been associated with poorer prognoses in tumors >2 cm, although pregnancy and perinatal outcomes are better compared to ART [4].

Minimally invasive surgery has faced scrutiny since the LACC trial, leading to restrictions in Japan. However, emerging trials like ConCerv, GOG278, and SHAPE indicate a trend toward less invasive surgeries for early-stage cervical cancer [7,8]. Nevertheless, JCOG 1101 suggests that modified radical hysterectomy may serve as an intermediary between simple and radical surgery. In our institution, laparoscopic lymph node dissection is performed to compensate for the shorter parametrium resection range in VRT. Our outcomes for VRT, both in terms of survival and fertility, are comparable to standard treatment [4,9,10]. Our institution has experience with over 100 cases of VRT, and 55 patients have successfully had children. Due to the limitations of available references, we have already published our own data [11]. We acknowledge that VRT for large tumors remains controversial. However, the recurrence rate after VRT at our institution is 5-6%, and the five-year survival rate for our patients is 99%. This indicates that even in cases of recurrence, patients can survive by transitioning to standard treatments such as additional surgery, radiation, or chemotherapy.

The reason why TC was chosen as chemotherapy is based on the JGOG0505 trial. TC and paclitaxel/cisplatin (TP) have been established as the standard of care in cervical cancer [12]. In this case, the patient spontaneously conceived and delivered a near-term baby, with a cervical cerclage placed during surgery to prevent infection. However, this approach may not be suitable for all patients, especially if NAC proves ineffective. Standard treatment should be considered promptly in such cases. While options like adoption or surrogacy exist globally for women without a uterus, fertility preservation remains limited in Japan. Uterine transplantation is being explored but is currently contraindicated in malignancies due to the requirement for immunosuppression. Future clinical trials, such as the CONTESSA trial [13], will help clarify the role of NAC in fertility preservation for patients with cervical cancer.

Considering the high recurrence rate associated with VRT, post-delivery follow-up includes monthly cervical cytology and sonography, as well as a CT scan every six months. There are currently no signs of recurrence.

## Conclusions

This case report highlights the successful management of cervical cancer using NAC followed by VRT. The lack of negative data on this type of treatment does not exclude the possibility that this case report represents an easy success story. While this approach has its limitations, the decision between standard treatment and fertility preservation must be personalized. If chemotherapy is not effective, standard treatment should be pursued. Fertility preservation may still be an option for patients with large tumors if NAC has successfully reduced the tumor size. Even with a high recurrence rate, early detection of recurrence allows for additional treatment, which can provide a prognosis similar to that of standard treatment. Therefore, post-treatment surveillance is crucial. We hope this report provides valuable insights into fertility-sparing treatments for cervical cancer.
